# Needs of Patients With Gynecologic Cancer and Their Caregivers for Obtaining mHealth-Supported Self-Management: Focus Group Study

**DOI:** 10.2196/48465

**Published:** 2024-10-03

**Authors:** Grace B Campbell, Hansol Kim, Tara C Klinedinst, Julie Klinger, Young Ji Lee, Heidi S Donovan

**Affiliations:** 1 School of Nursing Duquesne University Pittsburgh, PA United States; 2 Department of Obstetrics, Gynecology, and Reproductive Sciences School of Medicine University of Pittsburgh Pittsburgh, PA United States; 3 National Rehabilitation Research & Training Center on Family Support Office of the Senior Vice Chancellor of the Schools of the Health Sciences University of Pittsburgh Pittsburgh, PA United States; 4 Department of Rehabilitation Sciences University of Oklahoma Health Sciences Center Tulsa, OK United States; 5 Department of Internal Medicine OU-TU School of Community Medicine Tulsa, OK United States; 6 Department of Health and Community Systems School of Nursing University of Pittsburgh Pittsburgh, PA United States; 7 Department of BIomedical Informatics School of Medicine University of Pittsburgh Pittsburgh, PA United States

**Keywords:** gynecologic oncology, gynecologic cancer, self-management support, user-centered design, cancer distress, self-management, caregiver support, cancer information, women's health, family support, informal caregivers, informal care, mhealth

## Abstract

**Background:**

Family caregivers of individuals with gynecologic cancer experience high levels of distress. Web-based caregiver support interventions have demonstrated efficacy in improving caregiver outcomes. However, the lack of portability could be a limitation. Mobile health (mHealth) apps could fill this gap and facilitate communication between patient-caregiver dyads.

**Objective:**

We sought to obtain information on desired usage and features to be used to design an mHealth self-management support app targeting both patients with gynecologic cancer and their caregivers.

**Methods:**

We conducted Zoom focus groups with women who had been treated for gynecologic cancers (ovarian, fallopian, primary peritoneal, uterine, endometrial, cervical, and vulvar); patients were also asked to invite a self-identified “closest support person” (caregiver). A semistructured focus group guide was used to elicit information on patients’ and caregivers’ perceived gaps in information and support, desired features of an mHealth app, and interest in and preferences for app usage. After transcription, rapid qualitative analysis using a thematic matrix was used to identify common themes across groups.

**Results:**

A total of 8 groups were held. The final sample included 41 individuals with gynecologic cancer and 22 support persons or caregivers (total n=63). Patients were aged between 32 and 84 years, and most (38/41, 93%) were White and married. For caregivers (n=22), 15 (68%) identified as male and 7 (32%) as female, with ages ranging between 19 and 81 years. Overall, 59% (n=13) of caregivers were spouses. Questions geared at eliciting 3 a priori topics yielded the following themes: topic 1—gaps in information and support: finding relevant information is time-consuming; patients and caregivers lack confidence in deciding the urgency of problems that arise and from whom to seek information and guidance; topic 2—desired features of the mHealth app: patients and caregivers desire centralized, curated, trustworthy information; they desire timely recommendations tailored to specific personal and cancer-related needs; they desire opportunities to interact with clinical and peer experts through the app; and topic 3—interest and preferences for app usage: need for private space in the app for patients and caregivers to get information and support without the others’ knowledge; patients and caregivers desire having control over sharing of information with other family members.

**Conclusions:**

Designing a single mHealth app to be used by patients and caregivers presents unique challenges for intervention designers and app developers. Implications of the study suggest that app developers need to prioritize flexibility in app functionality and provide individuals the ability to control information sharing between patients and caregivers.

## Introduction

Caregivers of a family member with cancer experience high levels of distress and anxiety [1]. Caregivers of those with gynecologic cancer are particularly prone to high levels of distress [2], largely because these cancers are relatively rare, and for most (eg, ovarian, fallopian, and primary peritoneal cancers), no reliable screening exists. Thus, diagnosis is often not made until late stages, requiring intensive treatments with many side effects.

A growing literature documents the needs of caregivers of those with cancer in general and caregivers of those with gynecologic cancer in particular. Top-ranked needs include obtaining information about the cancer and treatment, finding ways to support individuals with gynecologic cancer, and maintaining their own health and well-being while providing care [3]. In busy gynecologic oncology practices where the focus is necessarily on treating the patient’s cancer, caregiver needs frequently are not prioritized or addressed [3], leaving caregivers without a dedicated support mechanism. Moreover, caregivers and patients exhibit a high degree of congruence regarding unmet needs for information and support [4].

A potential scalable solution for supporting individuals with cancer and their caregivers during treatment is the use of technology-based information, professional and/or peer support, and self-management coaching interventions. In particular, web-based self-management interventions that guide participants to develop their own plan of care, and monitor and manage their health proactively, are associated with positive effects on patient and caregiver well-being [5,6].

Our team has carried out a series of self-management support interventions based on the Representational Approach to Patient Education [7,8]; the WRITE Symptoms efficacy trial [9] among women with recurrent ovarian cancer; the SmartCare efficacy trial among caregivers of patients with a primary malignant brain tumor [10]; and a clinical implementation project to integrate family caregiver support into gynecologic oncology practice [11]. Each of these interventions followed the Representational Approach to guide patients or caregivers through self-management problem-solving. Key action steps include (1) representational assessment of symptoms or needs in the care situation; (2) identification of gaps, confusions, or misconceptions; (3) provision of targeted psychoeducation to address gaps in knowledge or correct misconceptions; (4) development of participant-generated goals and strategies to meet their goals; and (5) regular review of goal progress, strategy effectiveness, or barriers encountered and revision of goals and strategies as needed [9,12].

In the WRITE Symptoms 3-arm randomized clinical trial (N=497), the self-directed and nurse-delivered symptom self-management interventions (both computer mediated) were superior in improving patients’ symptom control compared to those receiving enhanced usual care at 8- and 12-week after baseline [13]. Furthermore, there was no difference in outcomes between the nurse-delivered and self-directed arms, and those in the self-directed arm were able to get through more symptoms more efficiently than those in the nurse-delivered arm. In the SmartCare randomized clinical trial, also based on the Representational Approach, caregivers of patients with primary malignant brain tumors receiving the SmartCare intervention reported significantly lower caregiving-specific distress and improved mastery over caregiving tasks compared to those receiving care as usual [10].

Despite the demonstrated benefits of web-based interventions in both patients with gynecologic cancer and in caregivers of individuals with primary malignant brain tumors, their lack of portability may be a limitation. Web-based interventions designed to be delivered via computer may be difficult to access during times of the most acute need. Mobile devices (eg, smartphones, tablets) could fill this gap. Such devices have become ubiquitous in American society: more than 70% of Americans use mobile devices (eg, smartphones) [14], and over 300,000 mobile health (mHealth) apps are available [15], presenting distinct scalability advantages over web-based interventions. Second, mobile platforms offer greater flexibility than computer web-based interventions for providing access to real-time feedback and resources [16]. Studies indicate that patients gain empowerment for managing their health and have positive health outcomes with the use of well-designed mHealth apps [17]. Translating web-based interventions into mHealth platforms presents challenges related to including key intervention ingredients in a mobile device, yet it presents opportunities to offer additional functionality and features not present in the original intervention.

As the first step in translating the SmartCare web-based intervention to an mHealth app, the research questions underlying this study were as follows: (1) What are the gaps in information and support in cancer care perceived by patients with cancer and family caregivers? (2) What are the features and functionality desired by patients and caregivers in an mHealth self-management support app? and (3) Would patients and caregivers use the mHealth app for day-to-day management, and if so, how would they prefer to use it (together or individually)? The objective of this study was therefore to obtain information on needed information and support, as well as desired usage and features, to inform the design of an mHealth self-management support app.

## Methods

### Sample and Setting

We recruited a convenience sample of women who had been treated for gynecologic cancers (ovarian, fallopian, primary peritoneal, uterine, endometrial, cervical, and vulvar) from a large quaternary care, university-affiliated health system in Western Pennsylvania. Women were also asked to invite a self-identified “closest support person” to participate. Using the health system’s honest broker system (HB015 University of Pittsburgh Medical Center [UPMC] Hillman Cancer Center, Pitt Biospecimen Core, and UPMC Enterprises), members of the cancer registry who had been treated for gynecologic cancer during the previous 5 years received a letter with information about the study from the Chair of the Division of Obstetrics, Gynecology, and Reproductive Sciences inviting interested individuals to participate. The invitation letter permitted recipients to define “family support person” as they wished; we did not specify a required relationship to an individual with cancer. We permitted individuals with cancer and family support people to participate in the same groups to permit identifying information needs and desired app features for both groups of users. Joint participation was also intended to elicit critical information about preferences for using the app individually or in partnership with the patient or caregiver.

### Ethical Considerations

The University of Pittsburgh’s Human Research Protections Office (institutional review board) approved this study as an exempt investigation (STUDY19110158). Participants provided informed consent to participate in the focus groups and were paid US $50 each.

### Procedure

Recruitment letters were prepared and mailed by registry staff to maintain confidentiality from research team members. Individuals interested in participating in the study after receiving the recruitment letter were asked to telephone the study coordinator to indicate interest in participating in the study.

A series of focus groups were conducted over 6 weeks using a secure, Health Insurance Portability and Accountability Act (HIPAA)–compliant Zoom account. Each group lasted approximately 90 minutes and was recorded using the built-in Zoom record feature. Patients and caregivers/support persons were scheduled for groups based on their convenience. The principal investigators (GBC and HSD) developed a semistructured focus group guide and conducted the focus groups to elicit information on the stated research questions. The focus group guide was designed specifically to elicit critical gaps in the information currently provided to patients with cancer and their families, desired features of a mobile app, and interest in and preference for an mHealth app for information and support.

After briefly sharing their cancer story to establish rapport, participants were asked about gaps in currently available information and support. Subsequently, they received a brief description of a potential mHealth information and support app and were asked about whether or not such an app would interest them; desired content, features, and functionality of such an app; and preferred ways of engaging with an app (ie, individually or in partnership with a caregiver/support person). Participants were also encouraged to verbalize any lack of interest in mHealth apps or in the potential content being discussed, to voice reasons for their disinterest, and for suggestions to make the app and content more appealing to them. A total of 8 groups were initially scheduled. The team debriefed after each focus group to identify any new information identified in each group. After the seventh group, no new themes had been identified. The eighth group was then held as scheduled; subsequently, the team agreed that saturation had been reached and no additional groups were scheduled. A priori topics and sample questions appear in Table 1.

**Table 1:** Sample focus group questions.

Topics	Sample focus group questions
Gaps in information and support	How do you currently obtain information and support for your cancer journey? Have you had difficulty obtaining the information and support you need? How might you use an app like this for your day-to-day information needs or support?
Desired features of a mobile app	If you are receiving most of your cancer care in your home community, how would you feel about a nurse from Pittsburgh—that is, not from your own community—reaching out to you to provide information and support? Tell us about reasons why you might not use such an app? Are there features or content that might make you more interested in it? Are there other things that you imagine that you might want to use it for, or other aspects or features that might make it more useful? Do you prefer to receive reminders to answer questions every several days, or would you rather answer questions only when you want to?
Interest in and preferences for a mobile app	Would you prefer to use the app yourself (meaning as a patient or a caregiver, you would use the app to manage your own most important concerns), or with your caregiver/patient together (meaning you would work on it together on shared goals)? Do you have thoughts about how that might work? This app would be a program that you could use on your own. You could also use it to get information and additional support from a nurse. How interesting would this be to you? Describe how you could see this app being used by the nurses or other staff in the clinic during the diagnosis and treatment process you experienced. Would you like a nurse to reach out to you after each time you answer questions through the app, or less frequently than that?

### Analysis

Each group’s recording was transcribed verbatim by the Qualitative Data Analysis Program at the University of Pittsburgh’s Center for Social and Urban Research. Individual speakers were neither identified nor delineated in transcripts to preserve the focus on the group, rather than on individuals, as the unit of analysis [18]. Following transcription, we used rapid qualitative analysis [19] to elicit thematic feedback from focus groups in a relatively short amount of time. Rapid qualitative analysis is a technique that uses a coding template initially developed from a subset of the data. The template is then expanded iteratively as additional themes are identified during coding of additional data and permits clustering of themes to help organize the data [20]. Rapid analysis thus provides a preliminary understanding of key themes, which can then be used to inform intervention development and implementation [21-23]. Initially, a preliminary codebook of themes was developed by the research team. Four investigators (HK, TK, HSD, and GBC) then independently coded the same transcript and compared agreement regarding transcript codes. The ReCal Reliability Calculator [24] was used to calculate intercoder reliability coefficients. Conflicts were adjudicated by the research team until agreement was achieved, and the preliminary codebook was finalized. Four team members then independently coded 2 more transcripts, and intercoder reliability coefficients were calculated for these 2 transcripts. We achieved a mean Cohen κ of 0.80 for these 2 transcripts. Following a common rapid qualitative analysis paradigm, once acceptable interrater reliability had been achieved, each remaining transcript was then coded by one validated coder. A thematic matrix was constructed using all raters’ identified themes to quickly identify common themes across focus groups. These themes will be used to inform the future design of app functionality and implementation strategies.

## Results

### Sample Characteristics

**Figure 1:**
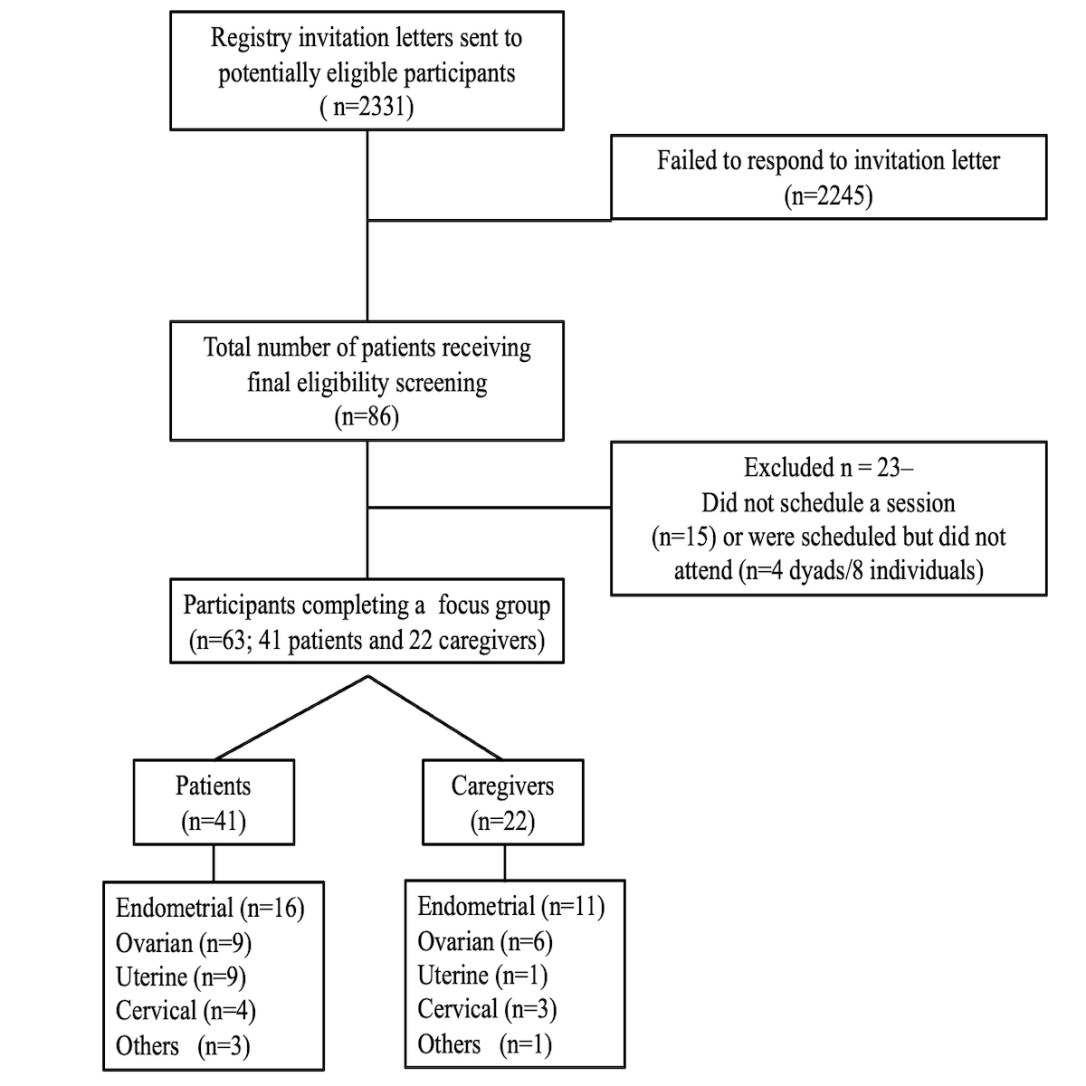
Study enrollment diagram.

A total of 86 individuals called the study coordinator after receiving an invitation to participate. Of those 86 individuals, 15 could not be reached further to be scheduled for a focus group. In total, 71 participants were ultimately scheduled to attend a group, although some (4 dyads, n=8) did not attend their scheduled session. The final sample included 41 individuals with gynecologic cancer and 22 family support persons/caregivers (total n=63). The focus group patients were aged between 32 and 84 years, and most (38/41, 93%) were White and married. Patients’ cancer diagnoses were endometrial (n=16, 39%), ovarian (n=9, 22%), uterine (n=9, 22%), cervical (n=4, 10%), and other (n=3, 7%). For caregivers (n=22), 15 (68%) individuals identified as male and 7 (32%) as female, with ages ranging between 19 and 81 years. Overall, 59% (n=13) of caregivers were spouses, followed by children, partners, siblings, and parents. Diagnoses of caregivers’ loved ones (patients) were endometrial (n=11, 50%), ovarian (n=6, 27%), uterine (n=1, 5%), cervical (n=3, 14%), and other (n=1, 5%). A recruitment diagram appears in Figure 1.

### Themes

**Figure 2:**
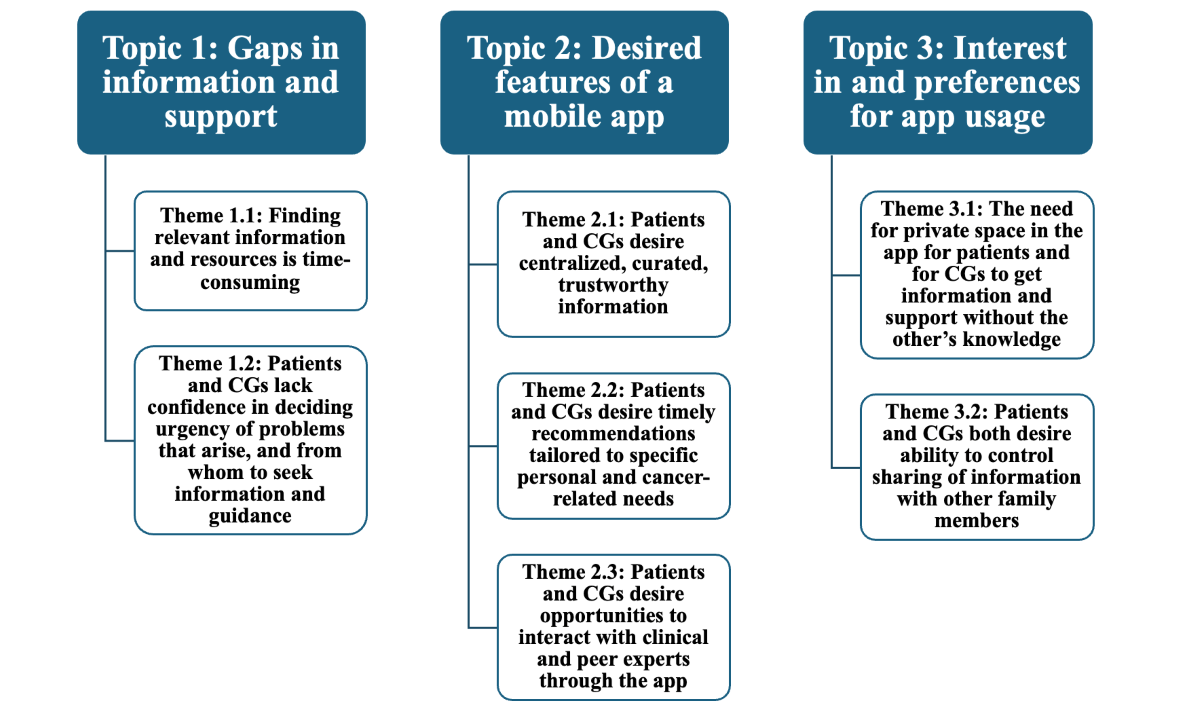
Topics and themes related to needs and preferences for support apps. CG: caregiver.

We identified 3 a priori overarching topics to guide focus group conversations (Gaps in information and support; Desired features of a mobile app; and Preferences for app usage). Topics and themes are depicted in Figure 2 and are discussed in detail below. Exemplar quotes that best embody the discussion across groups are provided.

### Topic 1: Current Gaps in Information and Support in Cancer Care

#### Theme 1.1: Finding Relevant Information and Resources is Time-Consuming

Patients and caregivers in all groups reported seeking information and formal support from their doctors and oncology nurses through phone calls, emails, or text messages when they were experiencing symptoms. Doctors and nurses provided assistance and support to patients who had medical questions. Yet, groups acknowledged still feeling a lack of information, so they sought information outside the clinical setting. One participant even noted that it felt like it was in her “own hands” to locate the help she needed. Participants simultaneously acknowledged that seeking support independently was time consuming, frustrating, and challenging. Consensus across groups suggests that much of the information that patients and caregivers found on their own had limited applicability to their specific situation, so they spent extensive time sifting through irrelevant information in search of something that might apply to them.

We spend a lot of time doing external research and kind of like trying to find, just, the information that kind of exists out there in the academic literature on the internet.

Patient, focus group 7

If there was a library that I knew I could log into and look up subjects...something dependable. Not something thrown on the internet. I don’t trust a lot of that stuff.

Patient, focus group 6

#### Theme 1.2: Patients and Caregivers Lack Confidence in Deciding Urgency of Problems That Arise, and From Whom to Seek Information and Guidance

Participants noted that when patients experienced new or concerning problems, such as worsening symptoms, it was difficult to self-triage—that is, to decide who to contact and where to find relevant resources. Patients and caregivers wanted to discuss their health concerns with their health care providers at the earliest onset of new symptoms. However, they were unclear as to what warranted an immediate phone call or visit to the clinic and what could be brought up at the next scheduled appointment. Despite their overall reluctance to seek web-based information, when making decisions about symptom urgency, many participants turn to searching for digital information. Examples from participants highlight how anxiety-provoking it could be to make these kinds of decisions in the middle of the night:

I’m just thinking—that at 2:30 in the morning, you look at something, and it specifically says, ‘Yes, this is something to be very concerned with,’ now you have to—what are you going to do at 3:00 in the morning?...That the level of anxiety is constantly being stirred, and it’s a challenge.

Caregiver, focus group 1

Finally, participants expressed needing extra reassurance and information about what was normal and not normal when dealing with cancer, particularly for family caregivers who may not have experience with cancer.

I would have liked to have known, 'Well, what do I—what happens if she’s bleeding? Or take her to the hospital’? Or, you know...the tiredness, it’s not something terrible, it’s something just happening.

Caregiver, focus group 8

### Topic 2: Desired Features of a Mobile App

#### Theme 2.1: Caregivers and Patients Desire Centralized, Curated, Trustworthy Information

According to participants, they were filled with uncertainty regarding the reliability of web-based information. Patients and caregivers spoke about turning to Facebook support groups, the internet, and library resources to obtain information about cancer (eg, nutrition, exercise programs, wigs, meditation, counseling services, etc). Yet many spoke of needing assurance that the information being provided is high quality, trustworthy, and curated by a team of gynecologic oncology experts. They acknowledged that what can be found on the internet is not necessarily helpful or true.

So it’s like, a place for medical science and psychology and social work, and all that, I think it’s important to engage librarians in all of this. Librarians are expert in assessing information, and compiling resources, so involve librarians.

Patient, focus group 3

I love the fact that there’ll be accurate supported research data information out there for the cancer patient and her support person.

Patient, focus group 1

One request was that the app provide glossaries of health terms with easily understood language. These glossaries would provide gynecological cancer-related information, such as chemotherapy agents and side effects, stages of cancer, symptom management, or medication and drug information, in a single place.

For the lay person, and make it easily understood, and they can apply it to what their pathology report, or what they learned at the doctor’s office. They have a lot to gain from knowing just the medical terminology, and the meaning behind it. People have no idea what that meant...So, like a glossary, I think, would be good.

Patient, focus group 4

Family caregivers also expressed a desire for anticipatory information on how to care for an individual with gynecologic cancer:

What do you expect during chemo? What do you expect during radiation? What can you do to help them through this process? That’s the information I think I would be most interested in through that app.

Caregiver, focus group 6

It should be noted that not all participants thought they would use the app, largely because of a lack of technological proficiency. Because of this, there was consensus that the information on an app would need to be accessible to all users, regardless of their level of technology skills. Participants also suggested that there should be alternative methods for those who are not tech-savvy to engage with needed information and support, such as a call center that can respond to calls or texts.

#### Theme 2.2: Patients and Caregivers Desire Timely Recommendations Tailored to Specific Personal and Cancer-Related Needs

Group participants, regardless of whether they were patients or caregivers, overwhelmingly voiced a desire for the app to provide specific information about cancer and treatment that is tailored, or “customizable,” to their place on the cancer trajectory. For example, participants who were early in the cancer journey wanted to receive information about the most common cancer symptoms, side effects of drugs and chemotherapy, alternative treatments, and nutritional information during treatment. Those who were further along the trajectory expressed the need for information regarding topics such as family genetic history and testing, as well as how to manage “survivor guilt,” the feeling that occurs when a person feels guilty after surviving a life-threatening situation while others they meet are not so fortunate.

There’s an immediate need for information when you’re first diagnosed, and then there’s a second tier of information after that of after you’ve gone through your surgery and your treatments. And then there’s a third level after that of, you’ve recovered...what are the information resources that help somebody be okay about the ongoing threat of cancer still being out there...That’s gotta be really rough. And so it requires a whole new range of information resources.

Patient, focus group 3

I was happy, and thankful, that I was recovering, and I only had three radiation treatments and no chemotherapy treatments. But my survivor guilt was bigtime...I’m the only one surviving this cancer. And I had a hard time, I still have a hard time with survivor’s guilt. So I think an app that would be customizable to survivor’s guilt would be good for me.

Patient, focus group 5

One topic for which no clear consensus emerged concerned built-in reminders (eg, to complete assessments and learning activities). Some group members noted that reminders would be especially helpful at certain times in the care trajectory, such as at diagnosis, because there are so many new things to remember and keep track of but less helpful during other times (eg, after treatment was completed and symptoms were stable). Those who endorsed reminders talked about the importance of reminders and personalized support for exercise, diet, and other activities related to recovery from cancer. They suggested that the app should send push notifications to remind patients of upcoming appointments or activities and offer personalized support based on their stage of recovery.

if it’s a push that’s coming out—it would be more interesting to me. It could then basically require me to respond, so that I would be able to say, ‘Yes, I’ve got the rash,’ or, ‘No, I haven’t, but my hair is falling out,’ or some other reaction.

Patient, focus group 4

Some even noted that the reminder could help them to talk with their partner in the cancer journey about topics of interest.

If, the same little reminder, or bit of information was being sent to both the patient and the caregiver, it might help to even open conversation. “Oh, did you see what the app sent on us our phone at lunch time today? What do you think about that?”

Patient, focus group 3

However, other respondents in our study noted that they would prefer not to have reminders, perceiving them to be intrusive because “Reminders...can come at awkward time...” or can cause unwanted emotions, especially during times when they are able to forget the cancer and go about their daily lives:

You’re pulling on emotions that’ve been subdued while you’re making the spaghetti.

Caregiver, focus group 1

Groups thus agreed that the ability for individual users to customize reminders according to what was most helpful to them is an important app feature. Flexibility to be able to change reminder frequency based on needs was also noted.

#### Theme 2.3: Patients and Caregivers Desire Opportunities to Interact With Clinical and Peer Experts Through the App

Timeliness was also reflected in the request by several dyads to be able to communicate with a health care professional via the mHealth app. A key stipulation was that the communication should be with someone knowledgeable about the type of cancer and treatment. Group members felt that professionals or providers did not need to be members of the patient’s own treatment team; however, they noted that it should be someone who is familiar with the cancer and the treatment trajectory for their particular type of cancer.

A nurse or somebody that you would be able to, like, reach out to and talk to...that’s an important aspect that should be included no matter what...So having somebody was empathetic, and understood the situation, and would be willing to talk with you and walk through questions. That would be an important thing. It would’ve been really appreciated by us.

Patient, focus group 7

They also recognized the potential of interacting with peers through the app. Participants noted that mHealth platforms could enable them to share concerns and experiences, as well as to receive both practical and emotional support from other cancer patients and their caregivers.

I like chat groups for some practical down-to-Earth advice, because those women went through what I’m going through... somebody from a chat room would say, you know, “Put a thick cream on it. That’s what helped me in the past.” That’s the kind of support I need.

Patient, focus group 2

If like maybe caregivers could connect to other caregivers, or patients could connect to other patients. I think it would be really nice if I could like post a question in a forum, or something...like someone could share an article with me, I think that would be really nice.

Patient, focus group 8

Their recommended formats for communication included question-and-answer or chat room features that can be monitored and moderated by health care providers to ensure accuracy, provide practical cancer advice, or share concerns.

### Topic 3: Interest in and Preferences for App Usage

#### Theme 3.1: The Need for Private Space in the App for Patients and for Caregivers to Get Information and Support Separately, Without the Other’s Knowledge

Consensus across groups indicates that most people would be interested in an app provided it met their previously voiced concerns regarding trustworthiness, efficiency, ability to tailor information gleaned, and ability to interact with peers and knowledgeable clinicians through the app. Both patients and caregivers resoundingly endorsed the need for a private place to commiserate with peers and to have autonomy and privacy from the other member of the dyad when seeking information. Patients and caregivers alike spoke about wanting a place to express themselves openly and confidentially, without concern for how the other might feel if they could see what was being shared.

One participant made the analogy of “separate rooms...to sit on comfy sofas:”

It would give the cancer survivor an opportunity to commiserate with other cancer survivors...Sometimes I think the cancer survivors just need a way to be able to express their trepidation and fears with like-minded other survivors. If it can have a separate room—a separate room where survivors can go to commiserate...to sit on comfy sofas digitally, and commiserate...to have an ‘adult tantrum!

Patient, focus group 3

Caregivers recognized the importance of giving patients privacy and autonomy to make their own decisions on what to share and what not to share. Patients similarly recognized that family members also need a private space to share frustrations or other emotions without the fear of upsetting the patient.

I was just thinking about my mother. She passed of cancer. [she] was very private, though. So she would not always want me to know what was going on, much to my frustration, but she does have that right...

Caregiver, focus group 6

And he [the caregiver] may want to share a grievance or a frustration, or, you know, ask a question, or need help, that he might not want me to know that he’s seeking out. ‘Cause he doesn’t want to upset me, or whatever. So I would want him to have autonomy in it also, so that he could feel comfortable sharing and saying what he needed without me being privy to it.

Patient, focus group 6

#### Theme 3.2: Patients and Caregivers Both Desire Ability to Control Sharing of Information With Other Family Members

Several participants mentioned that they would like the ability to share information with family members that they wished to discuss later. They spoke of using app topics as a way to open potentially difficult conversations with a family member. One patient commented on the ability to share articles and information with family caregivers to access at their own convenience:

I’d want to...send a link to my husband, or send a link to my daughter. Because when I want to [read information] might not be when they want to do it. So to sit down and say, “Okay, we have to do this together now,” would be burdensome. So I think it would be more helpful to have access themselves, then they can go look up whenever it’s convenient for each individual.

Patient, focus group 4

However, the desire to share information was far from universal; other participants spoke about the dynamic and changing nature of their desires for information sharing and wanting to have ultimate control. Some spoke of the ability to share different information with different support persons:

I think it would be helpful to have levels set up. You could designate this person has access to everything; this person has access to this amount of information.

Patient, focus group 4

Loss of privacy and control during cancer treatment was a strong theme that resonated with most participants. They felt that the app could empower them to control a small amount of privacy during a process that leaves many feeling as if they no longer have any privacy left:

Well—at one point I’m like, “I don’t need you to know everything,” [laughs]—I don’t need him to know that I’m not drinking, because then he’s gonna give me a hard time about it. [laughs] But then—I like the idea that your caregiver—your partner—would have the ability to get a snapshot, “How are you doing today.” I do appreciate that... Because there’s so much loss of privacy already going through this treatment, that like, “I have to tell you

providers] everything?”... you know what I mean? Like, “I have nothing for me?” Patient, focus group 7

## Discussion

### Principal Findings

In this study, we endeavored to identify gaps in information and support in cancer care perceived by patients with cancer and their caregivers; features and functionality desired by patients and caregivers in an mHealth self-management support app; and whether patients and caregivers would prefer to use an mHealth app for support together or individually. A novel finding is that patients and caregivers desire help in determining the urgency of symptoms and concerns in order to “self-triage” regarding whether and when to seek care. Patients and caregivers also want trustworthy, vetted, curated information and support to supplement the care that they receive from their clinicians, and this information should be tailored to their point in the treatment trajectory and to their preferences. Our results suggest that mHealth self-management support apps are a useful and acceptable way to receive such support, provided that specific needs, concerns, desired features, and customizability were included in the app. A second notable novel finding of this study is that both patients and caregivers each desire to have a space that is their own, private from the other, and they each desire to have control over what information about their symptoms and information seeking is shared with the other. We discuss implications of these novel findings for app designers below.

Among our sample of patients with gynecologic cancer, we found nearly universal agreement that getting appropriate, personalized information and support throughout the cancer care trajectory is time-consuming. Patients and caregivers note having spent an extreme amount of time searching for information without any assurance as to the quality of information they located. They also noted a sense of “information overload,” consistent with prior literature suggesting that the volume and complexity of internet cancer information is overwhelming [25], leaving them confused and overwhelmed [26]. One study found that 91% of web-based health information seekers either need or want navigational support to locate relevant information. This underscores the importance of designing our app to provide effective navigation for patients with gynecologic cancer and their caregivers [27].

In a novel finding, patients and caregivers expressed feelings of uncertainty as to how to self-triage; that is, they lacked confidence in determining whether a particular symptom warranted an immediate call to the provider or not. They articulated the need for a decision support aid for symptoms to help determine urgency and identify appropriate care, especially during hours when the oncology clinic is closed. These findings extend prior work on electronic support [28], which has primarily focused on treatment-related decision-making.

Designing a single mHealth app to be used by both patients and caregivers presents unique challenges for intervention designers and app developers. Results of this study highlight the need for flexibility in app functionality. Both patients and caregivers spoke of needing “a place of their own” to gather information and get peer and professional support without worrying or burdening their partner. Congruent with our findings, a recent systematic review [29] noted that patients desire the ability to control the sharing of information from health systems’ patient portals. Our findings extend this work by describing caregivers’ desires to similarly control the sharing of information about their concerns and information needs with their partner (the patient).

Privacy remains a key concern when designing sharing functionality for mHealth apps. Krebs and Duncan [30] found that 29% of US mobile phone users discontinue using mHealth apps due to lack of privacy stemming from apps sharing data with family members or friends. Our dyads noted that they specifically wanted the flexibility to share information and data when *they* chose to do so. Both caregivers and patients voiced the desire to maintain ultimate control over what is shared with their family members. Interestingly, these results contradict a recent study noting that patient and caregiver dyads preferred to use an official health system portal together, rather than individually [31]; this discrepancy could be because patients and caregivers may perceive our app as more personal and more focused on their individual needs, as opposed to a health system portal perceived as an extension of the hospital rather than as a personalized support space. Furthermore, our participants noted that the desire to share information through the app is not static—it may vary among patient or caregiver partners and may also vary over time as they move through the cancer trajectory. Thus, app developers will need to be cognizant of the need for flexibility in app functionality, allowing customization by users as often as desired.

### Implications

Our study highlights several key considerations for the development of mHealth apps to support patients with gynecologic cancer and their caregivers in self-management. As patients and caregivers struggle with finding relevant information and lack confidence in deciding the urgency of problems, future apps should prioritize providing easily accessible, reliable information tailored to individual needs. This can be achieved through the development of recommendation algorithms that streamline decision-making processes. While recommender algorithms have existed for years, current algorithms are primarily targeted toward clinicians rather than being patient centered [32,33]. Our findings can inform components that should be incorporated into the recommender algorithms to permit optimal customization for patients and caregivers.

Additionally, our study sheds light on the issue of data sharing between patients and caregivers. Despite ongoing debate about the HIPAA considerations involved in such data sharing [34], there is currently no policy supporting and clarifying data sharing in this context. Our findings can inform policy makers about the need for guidelines on information sharing and flexibility, especially through consumer-centered technology such as web-based or patient portals and mobile apps. This can help ensure that patients and caregivers have the necessary control over sharing information while receiving the support they need.

### Strengths and Limitations

Our study has several notable strengths and a few limitations that must be considered. Participants for this study were recruited from a cancer registry at a National Cancer Institute–designated cancer center housed within a university-affiliated tertiary care health system. The cancer center is comprised of urban, suburban, and rural satellite centers and serves individuals from a wide geographic area, yielding a diverse pool of potential participants. We targeted patients currently receiving treatment as well as those who may have completed cancer treatment up to 5 years in the past. This approach provided important perspective regarding the diverse and dynamic needs of cancer dyads throughout the care trajectory. Despite these strengths, respondents to the recruitment letter for this study were largely those who had received care at the urban campus, even though a number lived an hour or more outside the city. Such individuals may differ from those who choose to receive care in rural areas closer to their residence; these differences could reduce the applicability of our results to mHealth design for these individuals. Additionally, our study sample was 94% (59/63) White, reflecting a higher percentage of White individuals than in the overall region (63.8%) [35]. Generalizing our results to non-White and rural-dwelling individuals should therefore be done with caution, and future work should purposively sample for a more diverse sample.

Focus group participants were overwhelmingly positive about the care they had received through the gynecologic oncology practice and were eager to discuss their experiences. The group facilitators (GC and HSD) maintain a clinical affiliation at the gynecologic oncology clinic and were perceived as extensions of a place of trust by participants. Thus, a sense of openness was achieved quickly at each group session, leading to rich discussion related to unmet needs and suggestions for important features. Such an open discussion may not have been achieved in a focus group that was perceived to be conducted by researchers with little clinical benefit for participants. Despite this strength, it must be noted that the presence of both patients and caregivers in the same groups may have inhibited full disclosure of concerns, stresses, and feelings by some participants. Further, patients that attended without a caregiver may have felt reluctant to express opinions about their caregivers’ experiences, not wanting to be perceived as speaking for someone who was not present while in the presence of other caregivers. This potential limitation is congruent with our finding that universal sharing of information and concerns is not desirable, but that sharing controlled by each individual within their comfort level, would be a desirable app feature.

An important limitation concerns the timing of this study. Our focus groups were conducted during the COVID-19 pandemic, which may have contributed to our finding that most participants would appreciate an mHealth self-management support option. Because traditional options for face-to-face interaction with the health care team were limited during the pandemic, this may have driven participants’ desire for more and better information and opportunities for interaction with others through an app. Yet, the timing of this study could also be a strength: the salience of information and support shortcomings among cancer dyads during the pandemic and the resultant desire for more effective mHealth solutions may have provided deeper insights into this topic than we could have gained outside the pandemic.

### Conclusions

Randomized controlled trials have demonstrated that mHealth interventions encourage proactive self-management skills and improve well-being while reducing secondary disease complications and health care costs [36-38]. mHealth apps can also improve adherence to treatment regimens for chronic conditions [39] and can positively impact long-term self-management [40,41]. However, for mHealth interventions to achieve widespread use in real world clinical settings, app developers must focus on end users’ desired uses, features, and functionality. Our study provides novel input from potential end users regarding components of a self-management support app for dyads with cancer in a gynecologic oncology program that will spearhead development and testing of a future mobile app.

## Abbreviations

HIPAA: Health Insurance Portability and Accountability Act
mHealth: mobile health
UPMC: University of Pittsburgh Medical Center
